# Mental health symptoms following a pregnancy complicated by cardiovascular disease: scoping review

**DOI:** 10.1192/bjo.2026.12051

**Published:** 2026-07-24

**Authors:** Iris Becene, Stephanie Vartany, Alyssa Grimshaw, Sona Jasani

**Affiliations:** Department of Obstetrics, Gynecology and Reproductive Sciences, Yale School of Medicine, Yale University, New Haven, Connecticut, USA; Harvey Cushing/John Hay Whitney Medical Library, Yale University, New Haven, Connecticut, USA

**Keywords:** Perinatal psychiatry, cardiovascular disease, pregnancy, postpartum mental health, postpartum depression

## Abstract

**Background:**

Pre-existing cardiovascular disease (CVD) affects 1–4% of pregnancies in the USA and is associated with maternal morbidity. High-risk pregnancies with increased medical supervision are associated with poor postpartum mental health, although the prevalence in those with CVD is unclear.

**Aims:**

This scoping review aimed to synthesise estimates of post-pregnancy mental health outcomes among people with pre-existing CVD.

**Method:**

Cochrane Library, Ovid Medline, Ovid EMBASE, Scopus, Google Scholar and Web of Science Core Collection were searched to identify relevant published literature, using controlled vocabulary and keywords for post-pregnancy mental health and CVD in pregnancy. The search was not limited by publication type, year or study design. Studies that included an assessment of post-pregnancy depression, anxiety or post-traumatic stress disorder (PTSD) symptoms after a pregnancy complicated by pre-existing CVD were included.

**Results:**

Of the 1591 studies screened, 7 met the inclusion criteria. In patients with pre-existing CVD, 4 studies assessed depressive symptoms and found a prevalence of 10.4–32.2%; 2 studies assessed anxiety symptoms and found a prevalence of 22.0–28.5%; and 1 study assessed PTSD and found a prevalence of 26.4% for mild symptoms and 18.2% for moderate symptoms.

**Conclusions:**

The prevalence of post-pregnancy depression, anxiety and PTSD symptoms among people with pre-existing CVD may be similar to, or higher than, estimates from the general population. However, there was high heterogeneity in the included studies. More research is needed to determine if there is an increased risk of postpartum mental health disorders for pregnant people with pre-existing CVD.

Cardiovascular disease (CVD) is the leading cause of maternal mortality in the USA, affecting 1–4% of all domestic pregnancies annually.^1–4^ CVD can be congenital or acquired, and can exist before pregnancy (i.e. pre-existing) or manifest during the pregnancy or postpartum time frame. Both pre-existing CVD or CVD induced by pregnancy are classified as high-risk pregnancies that can present serious risks to obstetric patients because of the physiological and haemodynamic changes placed on the maternal cardiovascular system.^1,5^ Roughly 15% of pregnant patients with CVD will need to be admitted to hospital during their pregnancy because of heart failure, acute coronary syndrome, thromboembolic events or endocarditis.^6^ Additionally, health equity-related concerns like increased pregnancy-related cardiovascular complications in minority groups have been reported.^7–9^

In general, patients with high-risk pregnancies are at risk of poor mental health outcomes during pregnancy and beyond.^10–12,27,28^ Long-term effects of poor maternal mental health may also be associated with a greater risk of developing CVD, with one study noting a 36% increased risk of CVD up to 20 years later in patients with perinatal and postpartum depression (PPD) compared with those without depression.^13^ Also important is that mental health disorders commonly co-occur, with up to two-thirds of patients with perinatal depression having one or more comorbid psychiatric disorders.^14^ Limited literature exists examining the association between mental health conditions and patients with CVD in pregnancy. Qualitative research in patients with pre-existing CVD reveals that these patients may feel shame and guilt regarding their choice to be pregnant, may feel helpless if clinical symptoms worsen as the pregnancy progresses, worry about their own death or disability, worry about their infant’s health, worry about passing heritable conditions to their children and may feel dismissed by their provider, causing sadness and disappointment.^15^ In non-pregnant individuals, CVD and mental health conditions are comorbid conditions.^16^ CVD is thought to cause blood–brain barrier damage through endothelial dysfunction and chronic inflammation.^17^ Coronary artery disease has been found to increase risk of depression by 70%.^16^ Conversely, depression and anxiety have been found to increase risk of coronary artery disease and myocardial infarction by 30%.^18^ Among these studies, women and individuals of younger age were at highest risk for developing depression after CVD.^19^ Given this data, it is plausible that pregnant patients with CVD may be at higher risk of experiencing poor mental health from both biologic and psychologic stress.

Despite the lack of literature specific to CVD in pregnancy and mental health, research has examined mental health in high-risk pregnancies generally. High-risk pregnancies can include pre-existing conditions like obesity, pre-gestational diabetes and CVD, as well as pregnancy-associated conditions like preeclampsia, gestational diabetes, abnormal placentation or fetal growth conditions.^20^ In high-risk pregnancies, such as those complicated by preeclampsia, the prevalence of PPD symptoms may be as high as 27–44% when measured by the Edinburgh Postnatal Depression Scale (EPDS).^20–22^ In contrast, global estimates suggest that PPD affects around 17% of postpartum individuals.^23^ A more recent meta-analysis reported a slightly higher depression prevalence of 25% in the general postpartum population since the COVID-19 pandemic,^24^ and could reflect the distinct stressors that occurred during this time.^25^ Data from a meta-analysis of 102 studies suggest that the prevalence of clinical postpartum anxiety is around 15% in the general population.^26^ Estimates of postpartum anxiety among high-risk pregnancies are scarce in the literature, although studies have found that obstetric complications are associated with increased risk of postpartum anxiety.^27^ For postpartum post-traumatic stress disorder (PTSD), the estimated prevalence is around 3–4% in the general population, whereas research in at-risk populations, including those with obstetric complications or histories of abuse, estimates the prevalence to be around 19%.^28^

The exact aetiology of postpartum mental health conditions is unknown, but is likely a confluence of many factors. Hormonal fluctuations following delivery may contribute, especially in predisposed individuals facing concurrent psychosocial stressors.^29,30^ Independent risk factors include a prior history of depression or anxiety, lack of social support, economic pressure and pregnancy complications.^31^ Ineffective coping has also been associated with increased PPD and anxiety symptoms.^32^ Thus, the contributors of poor mental health in those with high-risk pregnancies are likely multifactorial. Social stressors such as more frequent medical visits, biologic contributors like increased disease or symptom severity resulting from the physiologic changes of pregnancy and psychological stressors related to adverse maternal or neonatal outcomes like preterm birth, emergency caesarean delivery, fetal growth abnormalities and low Apgar scores have all been noted in the literature.^10,11,30–33^

These multifactorial players do not occur *in silo* and likely modify the effects of each other. Thus, a reasonable framework to consider when exploring the relationship between mental health and any type of high-risk pregnancy is ‘allostatic load’. This concept as defined by McEwen and Stellar is described as ‘the strain on the body produced by systems under challenge and the changes in metabolism and wear and tear on a number of organs and tissues’.^37^ Allostatic load accounts for the cumulative impact of chronic stress and life events on health, and may be helpful in elucidating the complex relationship between high-risk pregnancies and poor postpartum mental health.^10,11,37–39^ High-risk pregnancies in general are associated with reduced quality of life, higher burden of stressors and decreased ability to cope,^10^ but limited knowledge exists specific to each high-risk condition. Of all these conditions, preeclampsia has been extensively studied in the literature, and these findings may help guide similar inquiries into other high-risk conditions. Data on preeclampsia suggest that PPD, anxiety and PTSD symptoms may be higher in these pregnancies,^29,30,33–35^ with suggested biologic mechanisms including alterations in cerebral function by inflammatory mediators from endothelial dysfunction.^40,41^ Associated psychological stressors of preeclampsia may include fears of death and maternal or fetal harm.^42^ The interplay between biologic and psychosocial factors noted in preeclampsia research is also likely to occur for other high-risk conditions, like CVD in pregnancy. Thus, research efforts on maternal mental health outcomes should attempt to elucidate the unique interplay of biologic and psychosocial factors relevant to each type of high-risk pregnancy.

A better understanding of the mental health conditions experienced by patients with CVD in pregnancy is needed. Factors such as the pathophysiology of disease processes, course during pregnancy, CVD diagnosis time frame (i.e. newly developed perinatally versus pre-existing), as well as psychosocial stressors, will likely impact the mental health experiences of this patient population. Existing research on preeclampsia, which is a CVD-associated condition that develops during pregnancy or postpartum, may not account for the true allostatic load of patients with pre-existing forms of CVD.^38^ Thus, our goal for this scoping review is to focus only on patients with pre-existing CVD and explore the mental health outcomes (diagnosis or symptoms) and possible associations with these after delivery. The primary aim is to identify the prevalence of post-pregnancy depression, anxiety and PTSD conditions and/or symptoms in patients with pre-existing CVD. Anxiety and depression were included because the American College of Obstetricians and Gynecologists explicitly recommends screening for depression and anxiety in the postpartum period.^43^ PTSD was included as pre-existing CVD may contribute to acute health events such as myocardial infarction or stroke, which are associated with PTSD in non-pregnant samples.^44,45^ Outcomes were not limited to the immediate prenatal period to allow for exploration in the impact of pre-existing CVD during pregnancy beyond the postpartum period. The secondary aim is to explore associations between CVD and one or all of the above conditions, as well as whether CVD disease severity influences poor mental health risk. CVD severity is defined according to the World Health Organization Risk Classification for Women with Pre-existing Cardiovascular Disease. Pregnancy-induced cardiovascular conditions, such as preeclampsia, are not included to control for the possible influence that chronicity of CVD diagnosis may have on postpartum mental health. We hypothesise that the prevalence of post-pregnancy depression, anxiety and PTSD conditions or symptoms will be higher in patients with pre-existing CVD, and that more severe forms of CVD will be associated with poor mental health outcomes.

## Method

### Study design

Initially, the plan for this project was to conduct a systematic review to evaluate the risk between pre-existing CVD during pregnancy and subsequent development of post-pregnancy depression, anxiety and PTSD. Because of the small number of studies and the heterogeneity between existing studies, the decision was made to pivot to conducting a scoping review to report on the prevalence of mental health burden as defined by post-pregnancy depression, anxiety and PTSD, any associational data between pre-existing CVD in pregnancy and post-pregnancy mental health burden, and whether the severity of pre-existing CVD in pregnancy is a modifying factor for post-pregnancy mental health disorders. This scoping review was conducted and reported in accordance with Levac et al’s recommendations for scoping review methodology and the JBI Manual for Evidence Synthesis, Preferred Reporting Items for Systematic Reviews and Meta-Analyses (PRISMA) 2020 and the PRISMA Extension for Scoping Reviews (Supplementary Table 1).^46,47^ The original study protocol is available on PROSPERO (CRDG42024581624), and the scoping review study protocol is available on Open Science Framework.^48^ Pre-existing CVD was defined by conditions described in the 2019 American College of Obstetricians and Gynecologists Practice Bulletin on pregnancy and heart disease and include the following:^1^ congenital malformations (patent ductus arteriosus, atrial septal defect, ventricular septal defect, coarctation of aorta, tetralogy of Fallot), valvular disease (mitral stenosis, aortic stenosis, bicuspid aortic valve), aortopathies (aortic dissection, aortic rupture, aortic dilation), arrhythmias, connective tissue disorder (Marfan syndrome, vascular Ehler’s Danlos syndrome), pulmonary arterial hypertension, cardiomyopathy (hypertrophic cardiomyopathy, peripartum cardiomyopathy that occurred in a previous pregnancy), heart failure and coronary artery disease.

The WHO risk classification system, used to classify heart diseases in pregnancy by risk of morbidity and mortality, was used to determine pre-existing CVD disease severity.^49^

### Eligibility criteria

The inclusion criteria for the scoping review includes the following:

- study presents original data;
- study assesses the prevalence of post-pregnancy depression, anxiety or PTSD diagnosis or symptoms in patients with pre-existing CVD in pregnancy (defined below)
  OR
  study compares the odds/risk of post-pregnancy depression, anxiety or PTSD diagnosis or symptoms in patients with pre-existing CVD to patients without pre-existing CVD in pregnancy;
- post-pregnancy depression, anxiety or PTSD symptoms or diagnosis will be defined by the presence of one or all of these mental health conditions according to each study. Both studies that define these conditions by an established medical diagnosis from a healthcare professional or ICD code, meeting criteria for symptoms based on the screening tool used as defined by study authors, or by patient self-report will be included.

The exclusion criteria for the scoping review includes the following.

- study is a review of the topic or a narrative review;
- study is a case report;
- study is a clinical trial testing treatment for CVD in pregnancy or post-pregnancy depression, anxiety or PTSD;
- study is a qualitative study that does not include a measure of prevalence of depression, anxiety or PTSD;
- study includes depression, anxiety or PTSD as a risk factor for CVD in pregnancy;
- study investigates fetal CVD as a risk factor for maternal depression, anxiety or PTSD.

### Search strategy

An exhaustive search of the literature was conducted by a medical research librarian (A.G.) in Cochrane Library, Google Scholar, Ovid APA PsycExtra, Ovid APA PsycINFO, Ovid EMBASE, Ovid Medline, Scopus and Web of Science Core Collection databases to find relevant articles published from the inception of each database to 23 December 2024. Databases were searched using a combination of keywords and controlled vocabulary for PPD, anxiety and stress disorders and CVD. The search was not limited by language, publication type or year (full search strategies available in Supplementary Table 2). The search was peer-reviewed by a second medical librarian using the Peer Review of Electronic Search Strategies.^50^ Forward and backward citation chasing was performed with CitationChaser to identify additional relevant studies not retrieved by the database search.^51^

### Study selection

Search results from all databases were imported into an Endnote 21 library. Duplicates were removed with the Yale Reference Deduplicator.^52^ The deduplicated results were then imported into Covidence, a systematic review software for screening and data extraction (Veritas Health Innovation, Melbourne, Australia; www.covidence.org). Reviewers I.B. and S.V. both screened all titles and abstracts of the studies for inclusion in full-text review. Both reviewers also screened all full-text review articles for inclusion in the scoping review. In the event of disagreement, the two reviewers met to discuss and resolve the disagreement, with a third reviewer, S.J., acting as a tie breaker.

### Data extraction

Data were extracted by reviewers I.B. and S.V., with a third reviewer S.J. acting as a tie breaker. We extracted the study design, year of publication, country, participant characteristics (average age, parity, gravidity, other relevant sociodemographic), type of CVD, diagnostic criteria for CVD, use of the WHO risk classification score, time since index pregnancy (if study is retrospective), pre-pregnancy psychiatric history, type of mental health outcome (depression, anxiety and PTSD), diagnostic criteria for each mental health outcome, and type and source of financial support.

### Synthesis of results

The prevalence of each mental health outcome was summarised across the studies. Associations between the WHO risk classification level and mental health outcomes were reported to evaluate the relationship between CVD severity and mental health outcomes. Results from analyses comparing mental health outcomes after pregnancies complicated by pre-existing CVD and pregnancies without pre-existing CVD were also reported. These findings were further expanded on by describing the prevalence of mental health disorders by type of pre-existing CVD (congenital versus acquired) and the measurement tools used to ascertain mental health disorders.

### Outcomes

The mental health outcomes included in this review were depression, anxiety and PTSD symptoms or diagnosis following a pregnancy complicated by pre-existing CVD. The primary outcome is the reported percentage of post-pregnancy depression, anxiety and PTSD symptoms or diagnosis in pregnancies complicated by pre-existing CVD. The cut-off values reported by the authors for each scale to meet the criteria for depression, anxiety or PTSD are provided. We use the word ‘postpartum’ to describe mental health outcomes assessed within the first year postpartum, and ‘post-pregnancy’ to describe mental health outcomes assessed any time after delivery. The secondary outcome of effect size for the association between pre-existing CVD in pregnancy and mental health outcomes (depression, anxiety or PTSD) is reported as a risk ratio, odds ratio or difference in means. The secondary outcome of the association between pre-existing CVD in pregnancy and WHO risk classification is reported as a risk ratio or odds ratio.

## Results

### Studies included

**Fig. 1:**
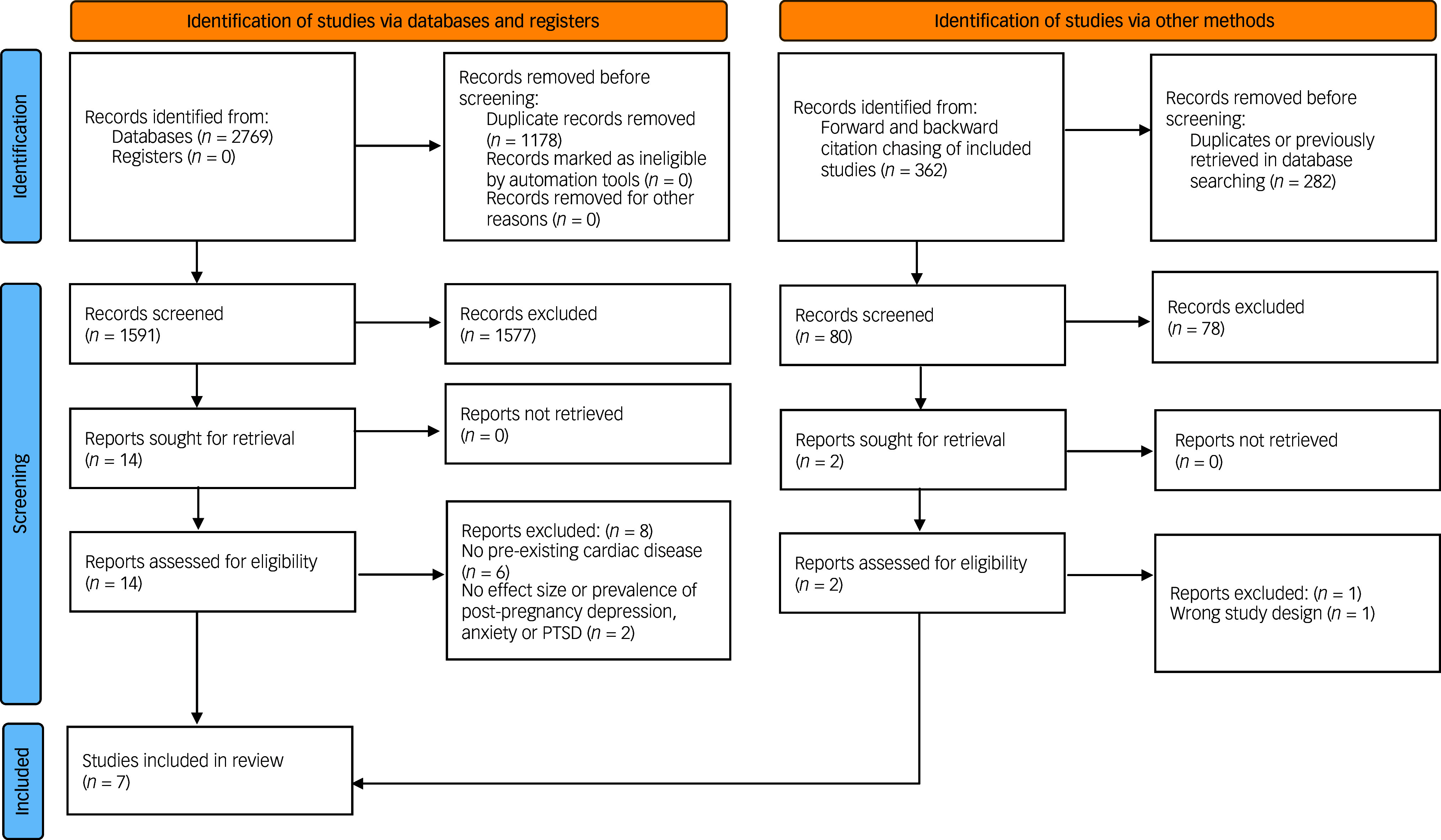
Fig. 1 long description. PRISMA flow diagram, adapted from Page et al, 2021.^53^ For more information, visit: http://www.prisma-statement.org/. PTSD, post-traumatic stress disorder.

The literature search yielded 2769 articles (Fig. 1). After removing duplicates, 1591 citations underwent title and abstract screening. Of these, 14 citations met the criteria for full-text review. Subsequently, six studies met the inclusion criteria for the study. An additional study was found through citation chasing, for a total of seven manuscripts included. We excluded nine studies for having no pre-existing cardiac conditions; no effect size or prevalence of post-pregnancy depression, anxiety or PTSD; or wrong study design (Supplementary Table 3).

### Characteristics of studies

Supplementary Table 4 provides an overview of the seven included studies. All studies were published within the past 11 years. Four studies were conducted with data from the USA,^54–57^ one from Germany,^58^ one from Australia^59^ and one from Canada.^60^ Five were cohort studies,^54–57,60^ one was a cross-sectional study^58^ and one was an exploratory descriptive study.^59^

Pre-existing CVD in pregnancy encompassed both acquired (i.e. hypertrophic cardiomyopathy, heart failure, coronary artery disease, valvular disease and arrhythmias) and congenital conditions (i.e. patent ductus arteriosus, atrial septal defect and ventricular septal defect). In the study by Panelli et al, the majority of those with acquired heart diseases were diagnosed before pregnancy, although some participants with arrhythmias were diagnosed in early pregnancy.^54^ Hutchens et al’s study included those with pre-existing CVD (*n* = 14) and those diagnosed during pregnancy (*n* = 6) or postpartum (*n* = 23).^59^ As this study reported the prevalence of post-pregnancy probable clinical anxiety among individuals with pre-existing CVD and presented probable clinical levels of depression and anxiety specifically for those with congenital or genetic heart disease, which are pre-existing conditions, it was included; however, only data pertaining to pre-existing, congenital or genetic disease were extracted. CVD diagnosis was ascertained by self-report,^56,59^ medical record abstraction^54,58^ and ICD codes.^57,60^

Post-pregnancy mental health conditions were most commonly measured with standardised questionnaires, although some studies abstracted mental health diagnoses from ICD codes or clinician diagnosis in the medical record.^57,60^ Post-pregnancy depressive symptoms were measured by various scales, including the EPDS,^54,58^ the Hospital Anxiety and Depression Scale (HADS),^58^ the Depression, Anxiety, and Stress Scales (DASS-21)^59^ and the Patient Health Questionnaire-9 (PHQ-9).^56^ Anxiety symptoms were measured by the DASS-21 and the HADS.^58,59^ PTSD symptoms were measured by the Impact of Event Scale – Revised (IES-R).^58^ Post-pregnancy mental health outcomes were assessed at various time points, including at 6 weeks postpartum,^54,56,57^ within 6 months postpartum^58^ and up to an average of 11 years postpartum.^59,60^

### Prevalence of post-pregnancy depression

The prevalence of post-pregnancy depressive symptoms among people who had a pre-existing CVD during pregnancy was reported by six studies and ranged from 10.4 to 32.2%. Of the two studies that used the EPDS to assess depressive symptoms, probable clinical levels of PPD ranged from 18.3 to 32.2%.^54,58^ One study used the HADS for assessing symptoms, indicating a prevalence of 14.9% for probable clinical levels of depression.^58^ One study used the DASS-21, and found that 14.0% of the congenital heart disease (CHD) sample met the criteria for probable current depression.^59^ One study used the PHQ-9 to assess postpartum depressive symptoms, with 10.4% of the sample screening positive for PPD. One abstract reported a PPD prevalence of 19%, although the method used to ascertain this prevalence was not reported.^55^ Post-pregnancy depression diagnosis and depressive symptoms prevalence ranged from 14.0 to 32.2% for congenital conditions,^55,58,59^ and was reported as 15.0% for acquired conditions in one study.^54^ Finally, post-pregnancy depression differed by time frame of diagnosis and included 6 weeks (five studies), 6–12 months (two studies) and an average of 5 years postpartum (one study). Loss to follow-up was reported in zero studies.

### Prevalence of post-pregnancy anxiety

The prevalence of probable clinical post-pregnancy anxiety among people who had a pre-existing CVD during pregnancy was reported by two studies, and ranged from 22 to 28.5%.^58,59^ One study used the HADS for assessing anxiety symptoms, with 24.8% of the sample meeting criteria for probable clinical levels of anxiety,^58^ whereas the other used the DASS-21 and found that 28.5% of those with pre-existing CVD had probable clinical levels of current anxiety and 22.0% of those with CHD had probable clinical levels of current anxiety.^59^ Anxiety symptom prevalence ranged from 22.0 to 24.8% for congenital conditions.^58,59^ No study reported the prevalence of probable clinical anxiety for acquired conditions diagnosed before pregnancy. Anxiety was assessed an average of 5 years postpartum^59^ and 11 years postpartum.^58^ Loss to follow-up was reported in zero studies.

### Prevalence of post-pregnancy PTSD

The prevalence of postpartum PTSD symptoms among people who had a pre-existing CVD during pregnancy was reported in only one study, and was 26.4% for mild symptoms and 18.2% for moderate symptoms.^58^ The IES-R was used to assess PTSD symptoms retrospectively to the postpartum period an average of 11 years postpartum. PTSD prevalence was reported only for congenital conditions. Loss to follow-up was not reported.

### Association between pre-existing CVD in pregnancy and post-pregnancy depression

Three studies analysed associations between pre-existing CVD and PPD symptoms or diagnosis, with none reporting significant associations in multivariate models.^56,57,60^ Past heart disease was associated with a 1.42 increased risk of PPD diagnosis in one study’s unadjusted model (risk ratio 1.42, 95% CI 1.36–1.49), although the association was not significant after adjusting for sociodemographic, medical and obstetric covariates, including number of clinic visits during pregnancy and length of hospital stay after delivery (risk ratio 1.05, 95% CI 1.00–1.11).^60^ Similarly, another study found that past heart conditions were not associated with screening positive for PPD after adjusting for sociodemographic, medical and obstetric covariates, although the association approached significance (odds ratio 1.92, 95% CI 0.98–3.79).^56^ A third study reported a statistically significant association between previous heart failure and hospital admission for PPD in a univariate model (odds ratio 13.9, 95% CI 3.5–55.9), but this association did not persist after adjusting for other chronic medical conditions, zip code income and type of insurance payer (odds ratio 6.9, 95% CI 0.3–158.0).^57^ This study also reported a statistically significant association between heart disease and hospital admission for PPD in a univariate model (odds ratio 5.7, 95% CI 2.1–15.3, *p* < 0.001), although this association did not persist in multivariate models (odds ratio 0.8, 95% CI 0.0–17.0). Similarly, a statistically significant association was found between atrial fibrillation and hospital admission for PPD in a univariate model (odds ratio 5.4, 95% CI 1.4–21.7, *p* = 0.017), which did not persist in multivariate models (odds ratio 4.6, 95% CI 0.2–103.1).

### Association between WHO risk classification for pre-existing CVD in pregnancy and post-pregnancy depression, anxiety and PTSD symptom prevalence

Three studies investigated trends between the WHO risk classification scores and PPD symptoms, with none reporting significant results.^54,55,58^ One study found that among those with probable PPD, there was a trend of more participants in the higher, or more severe, WHO risk classification score groups (of those with post-pregnancy depression, 26% in class 1, 35% with class 2, 35% with class 3 and 4% with class 4; of those without PPD (*n* = 103), 38% with class 1, 37% with class 2, 12% with class 3 and 8% with class 4), although this was not statistically significant.^54^ Another study reported that lower WHO risk classification scores were not associated with a greater risk of screening positive for PPD in univariate models (odds ratio 0.6, 95% CI 0.4–1.0) or multivariate models (adjusted odds ratio 0.7, 95% CI 0.4–1.3).^55^ Finally, a third study reported no correlation found between WHO risk classification scores and screening positive for PPD, which was measured retrospectively to the postpartum period an average of 11 years after delivery (*r*s = 0.148).^58^ This study was also the only study to investigate correlations between WHO risk classification scores and anxiety and PTSD.^58^ There was no association between WHO risk classification and probable clinical anxiety at the time of measurement (*r*s = 0.022), although there was a significant correlation between WHO risk classification and probable clinical PTSD, measured retrospectively to the postpartum period an average of 11 years after delivery (*r*s = 0.209, *p* = 0.031).

## Discussion

Among patients with pre-existing CVD in pregnancy, the overall prevalence of probable clinical symptoms was 10.4–32.2% for post-pregnancy depression, 22.0–28.5% for post-pregnancy anxiety, and 26.4% for mild or 18.2% for moderate post-pregnancy PTSD symptoms. Post-pregnancy depression was more commonly reported (seven studies), and post-pregnancy anxiety and PTSD were rarely reported (two studies and one study, respectively). Regarding post-pregnancy depression, the EPDS was the most used scale for determining probable clinical depression. The HADS and DASS-21 were both used for post-pregnancy anxiety symptom assessment and the IES-R was only used for post-pregnancy PTSD symptom assessment. Of the two studies investigating the association between pre-existing CVD in pregnancy and PPD symptoms or diagnosis, neither reported a significant association either initially^56^ or after adjustment for covariates.^60^ Additionally, one study reported no association between previous heart failure, heart disease or atrial fibrillation and hospital admission for PPD after adjustment for covariates.^57^ No studies investigated the association between pre-existing CVD in pregnancy and post-pregnancy anxiety or PTSD symptoms. Of the three studies reporting associations between WHO risk classification scores and PPD symptoms, none reported significant associations.^54,55,58^ One study investigated the correlation between WHO risk classification scores and post-pregnancy anxiety and PTSD symptoms, finding no association for anxiety and a significant association for PTSD symptoms.^58^

Our findings suggest that the prevalence of post-pregnancy depressive symptoms among individuals with pre-existing CVD may be comparable to the general postpartum population, although interpretation is limited by the small number of studies included in the review. Most studies (*n* = 3) reported PPD symptom prevalence to be 14–19%, which is similar to global estimates of PPD affecting 17% of postpartum individuals.^23,54,55,59^ In contrast, studies of high-risk pregnancies characterised by obstetric complications such as preeclampsia have reported the prevalence of PPD symptoms to be 27–44%.^20–22^ Two studies in this review, however, reported PPD symptom prevalences not consistent with estimates in the general population, with one reporting a lower prevalence (10.4%) and one reporting a higher prevalence (32.2%).^56,58^ The wide range in PPD symptom prevalence in this review is likely driven by substantial heterogeneity in study design, including differences in timing of assessment, screening tool utilised and classification of CVD.

The timing of measurement in relation to delivery may explain some of the variation in PPD symptom prevalence. Prevalence lowest (10.4%) in the only study that measured PPD symptoms prospectively within 6 weeks postpartum.^56^ In contrast, prevalence was highest (32.2%) in the study that assessed depressive symptoms retrospectively to the postpartum period an average of 11 years after delivery among individuals with CHD.^58^ Notably, the prevalence of depressive symptoms at the time of measurement was much lower (15%) in the same study.^58^ Evidence suggests that recalling depressive episodes is often inaccurate; only 40% of respondents with an episode of depression 10 years prior were able to accurately recall the episode in a study of non-pregnant individuals.^61^ Thus, this discrepancy in depressive symptom prevalence may be driven by recall bias. Although Hutchens et al’s study also measured depressive symptoms in the years following delivery, participants only reported symptoms at the time of measurement, with 14% of those with CHD screening positive for depression.^59^ Overall, the prevalence of PPD symptoms was highest when assessed retrospectively over a decade postpartum, followed by studies that retrospectively assessed PPD symptoms within the immediate postpartum period. Future studies should ideally implore a prospective cohort study design to accurately assess PPD symptom prevalence within 6 weeks postpartum, which is the recommended time frame to screen for PPD.^14^

Measures used to screen for depressive symptoms varied across the studies, which also may have contributed to the variation in probable depression prevalence. Depressive symptoms were highest (18–32%) among the two studies utilising the EPDS,^54,58^ followed by the study utilising the DASS-21 to assess current depressive symptoms several years postpartum (14%).^59^ The study utilising the PHQ-9 reported the lowest prevalence of PPD symptoms (10.4%).^56^ Conceptual differences in these screening tools may explain some of these differences. A network analysis study of over 2000 pregnant people found that the EPDS emphasises psychological symptoms (i.e. feeling sad or miserable) whereas the PHQ-9 prioritises the physical symptoms of depression (i.e. fatigue).^62^ Those with CVD may attribute their physical symptoms, such as fatigue, to their CVD rather than to their depression, which could potentially influence symptom reporting. However, no literature to date has investigated how measurement of depression differs across screening tools in individuals with CVD, making it difficult to understand whether this influenced our findings. In the perinatal population, however, evidence suggests that these instruments operate similarly in screening for PPD. A recent systematic review reported nearly identical operating characteristics between the PHQ-9 and EPDS in detecting perinatal depression.^23^ Similarly, the area under the curve (AUC) for the DASS-21 scale in identifying current depressive episode compared to DSM-IV criteria has been found to be similar to the AUC for the EPDS, at 0.76 *v*. 0.77, respectively.^63^ Therefore, the existing literature suggests that the PHQ-9, EPDS and DASS-21 similarly detect postpartum depressive symptoms.

Differences in screening thresholds between the two studies utilising the EPDS may also explain the variation in PPD symptom prevalence. In Freiberger et al’s study assessing PPD symptoms retrospectively a mean of 11 years postpartum, a cut-off of ≥9 was used as suggested by validation studies conducted among non-postpartum samples.^58^ In contrast, Panelli et al’s study assessed depressive symptoms within 6 weeks postpartum and thus utilised the validated cut-off of ≥10 to screen for PPD.^54^ As the prevalence of probable PPD was much higher in Freiberger et al’s study (32.2%) compared with Panelli et al’s study (18%), the lower cut-off value may explain why more cases of probable depression were detected. Current guidelines suggest utilising either the EPDS or the PHQ-9 to screen for PPD.^14^ These screening instruments have been found to have nearly identical operating characteristics, with similar sensitivities and specificities tools.^23,64^ Therefore, future studies investigating PPD after pregnancies complicated by pre-existing CVD should employ either the EPDS or the PHQ-9, using the cut-off values recommended for the study population.

Although all studies examined cases where CVD was pre-existing before pregnancy, the timing and nature of the diagnosis differed. In some studies, CVD was congenital and identified at birth or during childhood, whereas in others, it was acquired and diagnosed either years before pregnancy or just before conception. Additionally, some studies reported PPD symptom prevalences separately for congenital and acquired CVD, whereas others combined these conditions in their analyses. No clear pattern in post-pregnancy depressive symptom prevalence by CVD subtype emerged. Only one study compared the prevalence of depressive symptoms between those with congenital and pre-existing acquired diseases, reporting that 20% of those with congenital diseases screened positive for PPD symptoms compared with 15% of those with acquired conditions.^54^ There are distinct psychosocial stressors that may be experienced by those with CHD or pre-existing acquired CVD during pregnancy. For instance, individuals with congenital CVD may experience anxiety about transmitting their condition to their offspring, whereas those with acquired CVD may not face the same concern.^65^ Conversely, individuals with acquired CVD may struggle with adapting to a new diagnosis, a challenge that has been observed in studies of chronic illness among non-pregnant adults.^66^ Since pregnancy is often a significant life stressor, navigating it alongside a recent CVD diagnosis may further heighten vulnerability to postpartum mental illness. Given the potential implications of CVD classification and timing of diagnosis on postpartum mental health, future research on PPD should differentiate between congenital and pre-existing acquired CVD.

Of the two studies investigating the association between pre-existing CVD and PPD symptoms, neither found a significant association in adjusted models.^56,60^ It is important to note, however, that one study adjusted for length of stay at delivery and number of clinic visits during pregnancy, both of which remained significantly associated with PPD symptoms in adjusted models.^60^ These variables may reflect morbidity from CVD and thus may be mediators of the relationship between pre-existing CVD and PPD. For example, a study published using data from the National Inpatient Sample reported that pregnant people with CVD had significantly prolonged hospital stays compared with those without (3.9 *v*. 2.6 days, *p* < 0.001).^67^ Interestingly, another study in this review reported that participants with a positive PPD screen were significantly more likely to have had antepartum anticoagulation, blood transfusion and maternal–infant postpartum separation.^54^ Therefore, future studies should consider indicators of CVD morbidity as potential mediators when assessing the association between pre-existing CVD and PPD. Although the second study assessing the association between pre-existing CVD and PPD symptoms reported null findings, the association approached significance (adjusted odds ratio 1.92, 95% CI 0.98–3.79, *p* = 0.06) and thus may be detected in studies with greater power (*n* = 67 with CVD in this study).^56^ Consistent with these findings, an additional study examining heart disease in pregnancy and hospital admission for PPD also reported no association.^57^ However, interpretation is limited because hospital admission for PPD is rare, occurring in fewer than 1% of cases, and therefore does not accurately reflect PPD morbidity.^68^ Overall, more research is needed to determine if there is an association between pre-existing CVD during pregnancy and PPD. Future studies should ensure adequate statistical power with larger sample sizes and consider how markers of CVD morbidity may affect the relationship between pre-existing CVD and PPD. Additionally, no studies investigated the association between pre-existing CVD and post-pregnancy anxiety or PTSD, which warrants future research.

None of the three studies investigating WHO risk classification scores and PPD symptoms reported a significant association.^54,55,58^ Additionally, one study noted a trend toward higher WHO risk classification scores among those who screened positive for PPD,^54^ which is in contrast to another study that reported a trend toward lower WHO risk classification scores among those who screened positive for PPD.^55^ These conflicting results may be reflective of the limited number of participants with a WHO risk classification score of 4 (*n* = 21 total in the three studies).^1,54,55,58^ This category includes conditions contraindicated in pregnancy, such as pulmonary arterial hypertension, because of the extremely high risk of maternal mortality or severe morbidity during pregnancy, and current guidelines recommend pregnancy termination counselling for these patients.^1^ Future studies that include a greater number of participants with WHO risk classification scores 3 and 4 are needed to better understand the association between WHO risk classification scores and PPD.

Our post-pregnancy anxiety symptom prevalence are higher (24.8–28.5%) than the estimated global prevalence of postpartum anxiety of 12.3%, although estimates are limited to two studies.^58,59,69^ However, both studies assessed current anxiety symptoms an average of 5–11 years postpartum, indicating a gap in the literature on postpartum anxiety in this patient population. It is possible that our anxiety symptom prevalence results reflect higher baseline levels of anxiety found in individuals with both acquired and congenital CVD, which is often attributable to illness-related stressors.^70^ In both studies, the prevalence of current anxiety symptoms exceeded the prevalence of depressive symptoms, indicating that anxiety may be especially salient among those with pre-existing CVD.^58,59^ Qualitative research among pregnant individuals with pre-existing CVD has reported that many experience fears of cardiac deterioration and anxiety about the potential impact of their CVD on fetal health.^65^ Only one study investigated the association between WHO risk classification score and anxiety, reporting no significant association.^58^ As no studies assessed anxiety in the immediate postpartum period, there is a gap in the literature regarding the prevalence of anxiety following a pregnancy complicated by pre-existing CVD and the association between pre-existing CVD in pregnancy and anxiety. Future studies should ideally employ prospective cohort study designs, using validated perinatal anxiety measures such as the Generalised Anxiety Disorder-7 screener or the State-Trait Anxiety Inventory Short Form to fill this gap.^1^

Similarly, our postpartum PTSD symptom prevalence (18.2% for mild PTSD and 26.4% for moderate PTSD) is somewhat higher than what has been reported in the literature, although this result is limited to one study.^71^ For example, a recent meta-analysis reported that the prevalence of postpartum PTSD was estimated to be 3.1% in the general population and 15.7% in at-risk populations, defined by psychiatric history, trauma history and perinatal risk.^72^ It is important to note that the study included in our review measured PTSD symptoms retrospectively to the postpartum period an average of 11 years after delivery, which introduces the possibility of recall bias and thus may inflate symptom prevalence. However, this study also reported that higher WHO risk classification scores were associated with greater postpartum PTSD symptoms, suggesting that disease severity may affect traumatic experiences.^58^ Evidence from non-pregnant samples has found that CVD is associated with increased rates of PTSD, likely because of exposure to traumatic acute cardiac events.^73–75^ Qualitative research has revealed that those with pre-existing CVD during pregnancy fear the potentially life-threatening impact of their disease on their health and their child’s health.^65^ Given that the DSM-5 classifies PTSD as ‘exposure to actual or threatened death, serious injury or sexual violence to oneself, or others and subsequent symptoms’, it is plausible that experiencing a pregnancy complicated by pre-existing CVD may be a traumatic experience for some individuals and increase risk of developing PTSD.^76^ As only one study reported on this outcome, prospective studies are needed to assess the prevalence of postpartum PTSD in this population and to investigate the association between pre-existing CVD and postpartum PTSD. Future studies should use validated tools for assessing PTSD such as the Primary Care PTSD Screen, which is recommended for use in perinatal populations.^14^

This scoping review highlights substantial gaps in the literature regarding pre-existing CVD during pregnancy and post-pregnancy mental health outcomes, necessitating future research. Our recommendations for future studies are listed in Table 1. We recommend that future studies employ a prospective cohort study design to minimise recall bias and to accurately capture post-pregnancy mental health outcomes within the immediate postpartum period (6 weeks) and up to 1 year postpartum. Samples should include those with CHD and those with acquired CVD, to compare mental health outcomes between these groups, as they may experience unique psychosocial stressors.^66^ We also recommend investigating the association between pre-existing CVD diagnosis and mental health outcomes, exploring measures of disease morbidity as potential mediators of these relationships. Literature regarding PTSD and anxiety is especially limited in this patient population, and current evidence suggests that these mental health conditions may be especially salient. Future studies should employ either validated screening tools to capture symptom prevalence or utilise clinician diagnosis.

**Table 1:** Recommendations for future studies

(a)	Utilise a prospective cohort study design or assess symptoms within 6 weeks postpartum and up to 1 year
(b)	Include participants with both acquired and congenital CVD
(c)	Investigate anxiety and PTSD as postpartum mental health outcomes in addition to depression among those with pre-existing CVD
(d)	Use screening tools as recommended by regional obstetric oversight bodies, including: EPDS or PHQ-9 for depression GAD-7 or STAI-6 for anxiety PC-PTSD for PTSD
(e)	Stratify prevalence data of positive mental health screening or diagnoses by type of CVD (acquired versus congenital)
(f)	Assess the association between CVD severity (i.e. WHO Pregnancy Risk Classification) and postpartum mental health outcomes

CVD, cardiovascular disease; PTSD, post-traumatic stress disorder; EPDS, Edinburgh Postnatal Depression Scale; PHQ-9, Patient Health Questionnaire-9; GAD-7, Generalised Anxiety Disorder-7; STAI-6, 6-Item State–Trait Anxiety Inventory; PC-PTSD, Primary Care Posttraumatic Stress Disorder Screen; WHO, World Health Organization.

Few clinical recommendations can be made from this scoping review because of the limited evidence and high study heterogeneity. However, this review does highlight the importance of screening patients with CVD for mental illness in the postpartum period. Although current guidelines recommend universal screening for depression and anxiety in the postpartum period, real-world data suggests screening rates are as low as 3.0% for depression and 2.0% for anxiety.^14,77,78^ Screening for postpartum mental health conditions among those with CVD may be especially important, as postpartum mental illness have been found to increase CVD risk later in life. For example, PPD has been found to be associated with a 30–50% increased risk of hypertension, ischaemic heart disease and heart failure up to 20 years postpartum.^13^ For individuals already experiencing CVD morbidity, this increased risk may have compounding effects on future disease morbidity. To reduce this risk, current guidelines recommend integrating mental health specialists as needed into the prenatal care team for individuals with CVD during pregnancy and utilising community and national resources for support.^1,78^ Future research is needed to clarify whether preventive mental health support is necessary in this population and to identify the most effective strategies for integrating this care into clinical practice.

Our scoping review has several strengths. First, two independent reviewers screened all titles and abstracts and full texts for inclusion in the scoping review and two independent reviewers extracted all data included in the scoping review. A third reviewer was used as a tie breaker in the case of disagreements. Second, our broad inclusion criteria of multiple mental health outcomes (depression, anxiety and PTSD) and multiple time frames beyond the immediate postpartum period provides a thorough overview of the current literature on pre-existing CVD and postpartum mental health, allowing for identification of significant gaps in the literature. Third, our review was conducted in accordance with established scoping review methodology,^46,79^ with a pre-registered protocol on PROSPERO and additional scoping review registration on Open Science Framework.^48^ Finally, our literature search was conducted by a medical research librarian who ensured we captured studies not retrieved by database searches through citation chasing.

There are several limitations to this scoping review. First, we assessed a small number of studies, as only seven studies met criteria for inclusion in the scoping review. Second, there was high heterogeneity in study design among the included studies; studies relied on different screening tools (EPDS, PHQ-9, DASS, HADS) at different time points (6 weeks postpartum, 1 year postpartum, retrospectively 5 years postpartum, retrospectively 11 years postpartum), to assess depression, anxiety and PTSD symptoms. Third, two studies assessed postpartum mental health symptoms retrospectively an average of 5 and 11 years postpartum, introducing the possibility of significant recall bias. Fourth, only one study reported the prevalence of probable clinical depression for both those with congenital and pre-existing acquired CVD, limiting our ability to assess differences in depression by type of CVD. Fifth, only two studies reported results for anxiety and only one study reported results for PTSD, demonstrating a significant gap in literature. Sixth, four of the seven included studies had small sample sizes (*n* < 150), limiting their power to adequately detect statistically significant differences in mental health outcomes or provide representative estimates of probable clinical levels of depression, anxiety and PTSD.

Seventh, four of the seven studies included utilised data from the USA, which restricts generalisability of the findings to other countries. Finally, we did not evaluate study quality or risk of bias because of the high heterogeneity of study findings.

The literature examining pre-existing CVD during pregnancy and postpartum mental health disorders is limited. Future research examining depression, anxiety and PTSD in the postpartum period is needed among this patient population. Future studies should ideally employ a prospective cohort study design to evaluate the differing effects of acquired versus CHD and CVD severity. Postpartum mental health disorders should be assessed via validated screening tools or clinician diagnosis. As those with pre-existing CVD and mental illness are especially vulnerable to adverse cardiac events, it is vital to understand these risk factors in this population.

## Supporting information

10.1192/bjo.2026.12051.sm001 Becene et al. supplementary material Becene et al. supplementary material

## Data Availability

Data availability is not applicable to this article as no new data were created or analysed in this study.

## Supplementary material

The supplementary material is available online at https://doi.org/10.1192/bjo.2026.12051

## Fig. 1 Long description

The flowchart is divided into two main sections: Identification of studies via databases and registers, and Identification of studies via other methods. Each section outlines the steps taken to identify, screen, and include studies in the review. Panel A: Identification of studies via databases and registers. Records identified from databases total 2769. Records removed before screening include 1178 duplicates and 0 records marked as ineligible by automation tools. 1591 records are screened, with 1577 records excluded. 14 reports are sought for retrieval, and all 14 are assessed for eligibility. 8 reports are excluded for reasons such as no pre-existing cardiac disease or no effect size or prevalence of post-pregnancy depression, anxiety, or PTSD. 7 studies are included in the review. Panel B: Identification of studies via other methods. Records identified from forward and backward citation chasing of included studies total 362. Records removed before screening include 282 duplicates or previously retrieved in database searching. 80 records are screened, with 78 records excluded. 2 reports are sought for retrieval, and both are assessed for eligibility. 1 report is excluded for wrong study design. No reports are not retrieved in this section.

Navigate back to Fig. 1.
