# Supplementary material for: Mental health symptoms following a pregnancy complicated by cardiovascular disease: scoping review
Source: BJPsych Open. 2026 Jul 24;12(4):e197. doi: 10.1192/bjo.2026.12051 (PMC13419607; doi:10.1192/bjo.2026.12051)
Supplement: Becene et al. supplementary material [file S2056472426120511sup001.docx]

**Supplementary File**

Mental Health Outcomes Following a Pregnancy Complicated by Cardiovascular Disease: A Scoping Review

Iris Becene^1^, Stephanie Vartany^1^, Alyssa Grimshaw^2^, Sona Jasani^3^

^1^ Yale School of Medicine

^2^ Harvey Cushing/John Hay Whitney Medical Library, Yale University

^3^ 3Yale School of Medicine Department of Obstetrics, Gynecology and Reproductive Sciences

**Corresponding Author**

Iris Becene, BA

Yale School of Medicine

333 Cedar St, New Haven, CT 06510

iris.becene@yale.edu

**Supplementary Table 1: Reporting Guideline Checklists**

**Preferred Reporting Items for Systematic reviews and Meta-Analyses extension for Scoping Reviews (PRISMA-ScR) Checklist**

| **SECTION** | **ITEM** | **PRISMA-ScR CHECKLIST ITEM** | **REPORTED ON PAGE #** |
| --- | --- | --- | --- |
| **TITLE** | | | |
| Title | 1 | Identify the report as a scoping review. | 1 |
|  | | | |
| Structured summary | 2 | Provide a structured summary that includes (as applicable): background, objectives, eligibility criteria, sources of evidence, charting methods, results, and conclusions that relate to the review questions and objectives. | 1 |
|  | | | |
| Rationale | 3 | Describe the rationale for the review in the context of what is already known. Explain why the review questions/objectives lend themselves to a scoping review approach. | 2-4 |
| Objectives | 4 | Provide an explicit statement of the questions and objectives being addressed with reference to their key elements (e.g., population or participants, concepts, and context) or other relevant key elements used to conceptualize the review questions and/or objectives. | 3-5 |
|  | | | |
| Protocol and registration | 5 | Indicate whether a review protocol exists; state if and where it can be accessed (e.g., a Web address); and if available, provide registration information, including the registration number. | 4 |
| Eligibility criteria | 6 | Specify characteristics of the sources of evidence used as eligibility criteria (e.g., years considered, language, and publication status), and provide a rationale. | 4-5 |
| Information sources* | 7 | Describe all information sources in the search (e.g., databases with dates of coverage and contact with authors to identify additional sources), as well as the date the most recent search was executed. | 5 |
| Search | 8 | Present the full electronic search strategy for at least 1 database, including any limits used, such that it could be repeated. | 5 |
| Selection of sources of evidence† | 9 | State the process for selecting sources of evidence (i.e., screening and eligibility) included in the scoping review. | 5 |
| Data charting process‡ | 10 | Describe the methods of charting data from the included sources of evidence (e.g., calibrated forms or forms that have been tested by the team before their use, and whether data charting was done independently or in duplicate) and any processes for obtaining and confirming data from investigators. | 5-6 |
| Data items | 11 | List and define all variables for which data were sought and any assumptions and simplifications made. | 5-6 |
| Critical appraisal of individual sources of evidence§ | 12 | If done, provide a rationale for conducting a critical appraisal of included sources of evidence; describe the methods used and how this information was used in any data synthesis (if appropriate). | N/A |
| Synthesis of results | 13 | Describe the methods of handling and summarizing the data that were charted. | 6-8 |
| **RESULTS** | | | |
| Selection of sources of evidence | 14 | Give numbers of sources of evidence screened, assessed for eligibility, and included in the review, with reasons for exclusions at each stage, ideally using a flow diagram. | 7 |
| Characteristics of sources of evidence | 15 | For each source of evidence, present characteristics for which data were charted and provide the citations. | 7 |
| Critical appraisal within sources of evidence | 16 | If done, present data on critical appraisal of included sources of evidence (see item 12). | N/A |
| Results of individual sources of evidence | 17 | For each included source of evidence, present the relevant data that were charted that relate to the review questions and objectives. | 7-9 |
| Synthesis of results | 18 | Summarize and/or present the charting results as they relate to the review questions and objectives. | 7-9 |
|  | | | |
| Summary of evidence | 19 | Summarize the main results (including an overview of concepts, themes, and types of evidence available), link to the review questions and objectives, and consider the relevance to key groups. | 9-14 |
| Limitations | 20 | Discuss the limitations of the scoping review process. | 14 |
| Conclusions | 21 | Provide a general interpretation of the results with respect to the review questions and objectives, as well as potential implications and/or next steps. | 13-15 |
|  | | | |
| Funding | 22 | Describe sources of funding for the included sources of evidence, as well as sources of funding for the scoping review. Describe the role of the funders of the scoping review. | 11 |

JBI = Joanna Briggs Institute; PRISMA-ScR = Preferred Reporting Items for Systematic reviews and Meta-Analyses extension for Scoping Reviews.

*From:* Tricco AC, Lillie E, Zarin W, O'Brien KK, Colquhoun H, Levac D, et al. PRISMA Extension for Scoping Reviews (PRISMAScR): Checklist and Explanation. Ann Intern Med. 2018;169:467–473. [doi: 10.7326/M18-0850](http://annals.org/aim/fullarticle/2700389/prisma-extension-scoping-reviews-prisma-scr-checklist-explanation)

**Appendix Table 2: Search Strategies**

**Ovid Embase**

1 exp perinatal depression/

2 puerperal psychosis/

3 ((maternal or maternity or perinatal or antenatal or postnatal or prepartum or pre-natal or pre-partum or ante-natal or post-natal or puerperal or puerperium or post-partum or postpartum or peri-natal) adj3 (mental or depression or depressed or psychos* or psych* or stress* or distress* or anxiet* or anxious* or PTSD or sadness)).tw,kf.

4 ((baby or third-day or maternity or maternal) adj1 blues).tw,kf.

5 or/1-4

6 congenital heart malformation/

7 exp patent ductus arteriosus/

8 aortic coarctation/

9 Fallot tetralogy/

10 exp aortic stenosis/

11 exp Arrhythmias/

12 exp connective tissue disease/

13 Marfan syndrome/

14 exp Ehlers Danlos syndrome/

15 exp pulmonary hypertension/

16 exp aortic dissection/

17 exp aortic rupture/

18 exp cardiomyopathy/

19 exp heart failure/

20 exp coronary artery disease/

21 ((heart or cardiac or atrial-sept* or atrium-sept* or interatrial-sept* or inter-atrial-sept* or ventricle-sept* or ventricular-sept* or interventricular-sept*) adj3 (anomal* or defect* or malform* or ASD or perforat*)).tw,kf.

22 ((ductus or truncus) adj3 (arteriosus or arteriosis or botalli) adj3 (patenc* or persisten* or patent* or obliterat* or closure* or ligat*)).tw,kf.

23 ((persist* or patent*) adj3 ostium adj3 secundum).tw,kf.

24 ((atrial or interventricular or ventricul*) adj3 stunt*).tw,kf.

25 (cleft-heart-atrium* or Lutembacher* or membranous incomplete septum* or arrhythmi* or arrhytmi* or arrythmi* or disarrhythmi* or disarrhytmi* or disarrythmi* or dysarrhythmi* or dysarrhytmi* or dysarrythmi* or Ehlers Danlos).tw,kf.

26 (aort* adj3 (coarctatio* or dissect* or ruptur* or dilat*)).tw,kf.

27 (fallot adj3 (tetralogy or triology)).tw,kf. 14525

28 ((Mitral or bicuspid or left atrioventricular or aort*) adj3 (stenos* or re-stenos* or restenos* or regurgitat* or atresia*)).tw,kf.

29 (ectopic adj3 (rhythm* or beat*)).tw,kf.

30 ((heart or cardiac) adj3 (rhythm or beat) adj3 (problem* or disease* or disorder*)).tw,kf.

31 (Heart adj3 (aberrant or ventricle) adj3 (conduct* or contract*)).tw,kf.

32 ((connective tissue* or collagen*) adj3 (defect* or disease* or disorder* or dysplasi*)).tw,kf.

33 ((mesenchymal or sharp* or Weissenbach or Sjoegren or Sjogren or Sicca or mikulicz radecki or sacks) adj3 (disorder* or disease* or syndrome*)).tw,kf.

34 (CTD or MCTD).ti,ab.

35 (Marfan* adj3 (abiotroph* or syndrome* or disease*)).tw,kf.

36 ((pulmonar* or lung or portopulmonar* or porto-pulmonar*) adj3 (hypertens* or hemangiomatos* or haemangiomatos* or venoocclusive)).tw,kf.

37 (cor pulmonale or corpulmonale or eisenmenger* or aortopath*).tw,kf.

38 (right-heart adj3 failure*).tw,kf.

39 ((pulmonar* or lung) adj3 vascular adj3 (disease* or disorder* or failure*)).tw,kf.

40 (cardiomyopath* or heart myopath* or cardiac myopath* or myocardiopath* or primary myocardial disease*).tw,kf.

41 ((apical or familial or genetic or idiopathic or primary or non-obstructive or nonobstructive or obstructive) adj3 (HCM or HCMP)).tw,kf.

42 ((Beckwith or Weidemann or Costello or Danon or Fabry or Friedreich or LEOPARD or MELAS or MERRF or Noonan) adj3 (syndrome* or disease*)).tw,kf.

43 ((heart or cardiac or myocardial or cardiovascular or cardiogenic or cardiocirculatory or cardio-circulatory or cardiopulmonary or cardiorespiratory) adj3 (failure* or decompensat* or incompeten* or insufficien* or stand-still or standstill or shock or edema* or oedema*)).tw,kf.

44 ((diastolic or systolic or heart or ventricular or ventricle) adj3 (strain or overload or dysfunct*)).tw,kf.

45 ((LV or LVOT or RV or RVOT) adj3 obstruct*).tw,kf.

46 (coronary adj3 (disease* or syndrome* or aneurysm* or anomal* or malformat* or ectasia* or fistula* or calcificat* or calcified or constrict* or vasoconstrict* or spasm* or dissect* or perforat* or thrombos* or atherioscler* or athero-sclero* or atheroma* or arterioscler* or arterio-scler* or atherogene* or athero-gene* or atheros* or microatheros* or fibroatheroma* or fatty streak* or scleros* or cardioscleros* or bifurcation lesion* or occlus* or obstruct* or stenos* or restenos* or syndrome*)).tw,kf.

47 (Bland adj3 Garland adj3 White adj3 syndrome*).tw,kf.

48 ((allergic angina or Kounis) adj3 syndrome*).tw,kf.

49 no-reflow-phenomenon.tw,kf.

50 (CAD or ASCVD).ti,ab.

51 or/5-50

52 4 and 37

**Ovid MEDLINE(R) ALL**

1 Depression, Postpartum/

2 ((maternal or maternity or perinatal or antenatal or postnatal or prepartum or pre-natal or pre-partum or ante-natal or post-natal or puerperal or puerperium or post-partum or postpartum or peri-natal) adj3 (mental or depression or depressed or psychos* or psych* or stress* or distress* or anxiet* or anxious* or PTSD or sadness)).tw,kf.

3 ((baby or third-day or maternity or maternal) adj1 blues).tw,kf.

4 1 or 2 or 3

4 Heart Defects, Congenital/

5 Ductus Arteriosus, Patent/

6 Aortic Coarctation/

7 "Tetralogy of Fallot"/

8 exp Aortic Valve Stenosis/

9 exp Arrhythmias, Cardiac/

10 exp Connective Tissue Diseases/

11 Marfan Syndrome/

12 exp Ehlers-Danlos Syndrome/

13 exp Hypertension, Pulmonary/

14 exp Aortic Dissection/

15 Aortic Rupture/

16 exp Cardiomyopathies/

17 exp Heart Failure/ 160214

18 Coronary Artery Disease/ 82352

19 ((heart or cardiac or atrial-sept* or atrium-sept* or interatrial-sept* or inter-atrial-sept* or ventricle-sept* or ventricular-sept* or interventricular-sept*) adj3 (anomal* or defect* or malform* or ASD or perforat*)).tw,kf.

20 ((ductus or truncus) adj3 (arteriosus or arteriosis or botalli) adj3 (patenc* or persisten* or patent* or obliterat* or closure* or ligat*)).tw,kf.

21 ((persist* or patent*) adj3 ostium adj3 secundum).tw,kf.

22 ((atrial or interventricular or ventricul*) adj3 stunt*).tw,kf.

23 (cleft-heart-atrium* or Lutembacher* or membranous incomplete septum* or arrhythmi* or arrhytmi* or arrythmi* or disarrhythmi* or disarrhytmi* or disarrythmi* or dysarrhythmi* or dysarrhytmi* or dysarrythmi* or Ehlers Danlos).tw,kf.

24 (aort* adj3 (coarctatio* or dissect* or ruptur* or dilat*)).tw,kf.

25 (fallot adj3 (tetralogy or triology)).tw,kf. 14525

26 ((Mitral or bicuspid or left atrioventricular or aort*) adj3 (stenos* or re-stenos* or restenos* or regurgitat* or atresia*)).tw,kf.

27 (ectopic adj3 (rhythm* or beat*)).tw,kf.

28 ((heart or cardiac) adj3 (rhythm or beat) adj3 (problem* or disease* or disorder*)).tw,kf.

29 (Heart adj3 (aberrant or ventricle) adj3 (conduct* or contract*)).tw,kf.

30 ((connective tissue* or collagen*) adj3 (defect* or disease* or disorder* or dysplasi*)).tw,kf.

31 ((mesenchymal or sharp* or Weissenbach or Sjoegren or Sjogren or Sicca or mikulicz radecki or sacks) adj3 (disorder* or disease* or syndrome*)).tw,kf.

32 (CTD or MCTD).ti,ab.

33 (Marfan* adj3 (abiotroph* or syndrome* or disease*)).tw,kf.

34 ((pulmonar* or lung or portopulmonar* or porto-pulmonar*) adj3 (hypertens* or hemangiomatos* or haemangiomatos* or venoocclusive)).tw,kf.

35 (cor pulmonale or corpulmonale or eisenmenger* or aortopath*).tw,kf.

36 (right-heart adj3 failure*).tw,kf.

37 ((pulmonar* or lung) adj3 vascular adj3 (disease* or disorder* or failure*)).tw,kf.

38 (cardiomyopath* or heart myopath* or cardiac myopath* or myocardiopath* or primary myocardial disease*).tw,kf.

39 ((apical or familial or genetic or idiopathic or primary or non-obstructive or nonobstructive or obstructive) adj3 (HCM or HCMP)).tw,kf.

40 ((Beckwith or Weidemann or Costello or Danon or Fabry or Friedreich or LEOPARD or MELAS or MERRF or Noonan) adj3 (syndrome* or disease*)).tw,kf.

41 ((heart or cardiac or myocardial or cardiovascular or cardiogenic or cardiocirculatory or cardio-circulatory or cardiopulmonary or cardiorespiratory) adj3 (failure* or decompensat* or incompeten* or insufficien* or stand-still or standstill or shock or edema* or oedema*)).tw,kf.

42 ((diastolic or systolic or heart or ventricular or ventricle) adj3 (strain or overload or dysfunct*)).tw,kf.

43 ((LV or LVOT or RV or RVOT) adj3 obstruct*).tw,kf.

44 (coronary adj3 (disease* or syndrome* or aneurysm* or anomal* or malformat* or ectasia* or fistula* or calcificat* or calcified or constrict* or vasoconstrict* or spasm* or dissect* or perforat* or thrombos* or atherioscler* or athero-sclero* or atheroma* or arterioscler* or arterio-scler* or atherogene* or athero-gene* or atheros* or microatheros* or fibroatheroma* or fatty streak* or scleros* or cardioscleros* or bifurcation lesion* or occlus* or obstruct* or stenos* or restenos* or syndrome*)).tw,kf.

45 (Bland adj3 Garland adj3 White adj3 syndrome*).tw,kf.

46 ((allergic angina or Kounis) adj3 syndrome*).tw,kf.

47 no-reflow-phenomenon.tw,kf.

48 (CAD or ASCVD).ti,ab.

49 or/5-49

50 4 and 49

**Ovid APA PsycInfo**

1 postpartum depression/

2 postpartum psychosis/

3 ((maternal or maternity or perinatal or antenatal or postnatal or prepartum or pre-natal or pre-partum or ante-natal or post-natal or puerperal or puerperium or post-partum or postpartum or peri-natal) adj3 (mental or depression or depressed or psychos* or psych* or stress* or distress* or anxiet* or anxious* or PTSD or sadness)).mp.

4 ((baby or third-day or maternity or maternal) adj1 blues).mp.

5 or/1-4

5 exp Cardiovascular Disorders/

6 ((heart or cardiac or atrial-sept* or atrium-sept* or interatrial-sept* or inter-atrial-sept* or ventricle-sept* or ventricular-sept* or interventricular-sept*) adj3 (anomal* or defect* or malform* or ASD or perforat*)).mp.

7 ((ductus or truncus) adj3 (arteriosus or arteriosis or botalli) adj3 (patenc* or persisten* or patent* or obliterat* or closure* or ligat*)).mp.

8 ((persist* or patent*) adj3 ostium adj3 secundum).mp.

9 ((atrial or interventricular or ventricul*) adj3 stunt*).mp.

10 (cleft-heart-atrium* or Lutembacher* or membranous incomplete septum* or arrhythmi* or arrhytmi* or arrythmi* or disarrhythmi* or disarrhytmi* or disarrythmi* or dysarrhythmi* or dysarrhytmi* or dysarrythmi* or Ehlers Danlos).mp.

11 (aort* adj3 (coarctatio* or dissect* or ruptur* or dilat*)).mp.

12 (fallot adj3 (tetralogy or triology)).mp.

13 ((Mitral or bicuspid or left atrioventricular or aort*) adj3 (disease* or stenosis)).mp.

14 (ectopic adj3 (rhythm* or beat*)).mp.

15 ((heart or cardiac) adj3 (rhythm or beat) adj3 (problem* or disease* or disorder*)).mp.

16 (Heart adj3 (aberrant or ventricle) adj3 (conduct* or contract*)).mp.

17 ((connective tissue* or collagen*) adj3 (defect* or disease* or disorder* or dysplasi*)).mp.

18 ((mesenchymal or sharp* or Weissenbach or Sjoegren or Sjogren or Sicca or mikulicz radecki or sacks) adj3 (disorder* or disease* or syndrome*)).mp.

19 (CTD or MCTD).ti,ab.

20 (Marfan* adj3 (abiotroph* or syndrome* or disease*)).mp.

21 ((pulmonar* or lung or portopulmonar* or porto-pulmonar*) adj3 (hypertens* or hemangiomatos* or haemangiomatos* or venoocclusive)).mp.

22 (cor pulmonale or corpulmonale or eisenmenger* or aortopath*).mp.

23 (right-heart adj3 failure*).mp.

24 ((pulmonar* or lung) adj3 vascular adj3 (disease* or disorder* or failure*)).mp.

25 (cardiomyopath* or heart myopath* or cardiac myopath* or myocardiopath* or primary myocardial disease*).mp.

26 ((apical or familial or genetic or idiopathic or primary or non-obstructive or nonobstructive or obstructive) adj3 (HCM or HCMP)).tw,kf.

27 ((Beckwith or Weidemann or Costello or Danon or Fabry or Friedreich or LEOPARD or MELAS or MERRF or Noonan) adj3 (syndrome* or disease*)).tw,kf.

28 ((heart or cardiac or myocardial or cardiovascular or cardiogenic or cardiocirculatory or cardio-circulatory or cardiopulmonary or cardiorespiratory) adj3 (failure* or decompensat* or incompeten* or insufficien* or stand-still or standstill or shock or edema* or oedema*)).tw,kf.

29 ((diastolic or systolic or heart or ventricular or ventricle) adj3 (strain or overload or dysfunct*)).tw,kf.

30 ((LV or LVOT or RV or RVOT) adj3 obstruct*).tw,kf.

31 (coronary adj3 (disease* or syndrome* or aneurysm* or anomal* or malformat* or ectasia* or fistula* or calcificat* or calcified or constrict* or vasoconstrict* or spasm* or dissect* or perforat* or thrombos* or atherioscler* or athero-sclero* or atheroma* or arterioscler* or arterio-scler* or atherogene* or athero-gene* or atheros* or microatheros* or fibroatheroma* or fatty streak* or scleros* or cardioscleros* or bifurcation lesion* or occlus* or obstruct* or stenos* or restenos* or syndrome*)).tw,kf.

32 (Bland adj3 Garland adj3 White adj3 syndrome*).tw,kf.

33 ((allergic angina or Kounis) adj3 syndrome*).tw,kf.

34 no-reflow-phenomenon.tw,kf.

35 (CAD or ASCVD).ti,ab.

36 or/6-35

37 5 and 36

**Ovid APA PsycExtra**

1 postpartum depression/

2 postpartum psychosis/

3 ((maternal or maternity or perinatal or antenatal or postnatal or prepartum or pre-natal or pre-partum or ante-natal or post-natal or puerperal or puerperium or post-partum or postpartum or peri-natal) adj3 (mental or depression or depressed or psychos* or psych* or stress* or distress* or anxiet* or anxious* or PTSD or sadness)).mp.

4 ((baby or third-day or maternity or maternal) adj1 blues).mp.

5 or/1-4

5 exp Cardiovascular Disorders/

6 ((heart or cardiac or atrial-sept* or atrium-sept* or interatrial-sept* or inter-atrial-sept* or ventricle-sept* or ventricular-sept* or interventricular-sept*) adj3 (anomal* or defect* or malform* or ASD or perforat*)).mp.

7 ((ductus or truncus) adj3 (arteriosus or arteriosis or botalli) adj3 (patenc* or persisten* or patent* or obliterat* or closure* or ligat*)).mp.

8 ((persist* or patent*) adj3 ostium adj3 secundum).mp.

9 ((atrial or interventricular or ventricul*) adj3 stunt*).mp.

10 (cleft-heart-atrium* or Lutembacher* or membranous incomplete septum* or arrhythmi* or arrhytmi* or arrythmi* or disarrhythmi* or disarrhytmi* or disarrythmi* or dysarrhythmi* or dysarrhytmi* or dysarrythmi* or Ehlers Danlos).mp.

11 (aort* adj3 (coarctatio* or dissect* or ruptur* or dilat*)).mp.

12 (fallot adj3 (tetralogy or triology)).mp.

13 ((Mitral or bicuspid or left atrioventricular or aort*) adj3 (disease* or stenosis)).mp.

14 (ectopic adj3 (rhythm* or beat*)).mp.

15 ((heart or cardiac) adj3 (rhythm or beat) adj3 (problem* or disease* or disorder*)).mp.

16 (Heart adj3 (aberrant or ventricle) adj3 (conduct* or contract*)).mp.

17 ((connective tissue* or collagen*) adj3 (defect* or disease* or disorder* or dysplasi*)).mp.

18 ((mesenchymal or sharp* or Weissenbach or Sjoegren or Sjogren or Sicca or mikulicz radecki or sacks) adj3 (disorder* or disease* or syndrome*)).mp.

19 (CTD or MCTD).ti,ab.

20 (Marfan* adj3 (abiotroph* or syndrome* or disease*)).mp.

21 ((pulmonar* or lung or portopulmonar* or porto-pulmonar*) adj3 (hypertens* or hemangiomatos* or haemangiomatos* or venoocclusive)).mp.

22 (cor pulmonale or corpulmonale or eisenmenger* or aortopath*).mp.

23 (right-heart adj3 failure*).mp.

24 ((pulmonar* or lung) adj3 vascular adj3 (disease* or disorder* or failure*)).mp.

25 (cardiomyopath* or heart myopath* or cardiac myopath* or myocardiopath* or primary myocardial disease*).mp.

26 ((apical or familial or genetic or idiopathic or primary or non-obstructive or nonobstructive or obstructive) adj3 (HCM or HCMP)).tw,kf.

27 ((Beckwith or Weidemann or Costello or Danon or Fabry or Friedreich or LEOPARD or MELAS or MERRF or Noonan) adj3 (syndrome* or disease*)).tw,kf.

28 ((heart or cardiac or myocardial or cardiovascular or cardiogenic or cardiocirculatory or cardio-circulatory or cardiopulmonary or cardiorespiratory) adj3 (failure* or decompensat* or incompeten* or insufficien* or stand-still or standstill or shock or edema* or oedema*)).tw,kf.

29 ((diastolic or systolic or heart or ventricular or ventricle) adj3 (strain or overload or dysfunct*)).tw,kf.

30 ((LV or LVOT or RV or RVOT) adj3 obstruct*).tw,kf.

31 (coronary adj3 (disease* or syndrome* or aneurysm* or anomal* or malformat* or ectasia* or fistula* or calcificat* or calcified or constrict* or vasoconstrict* or spasm* or dissect* or perforat* or thrombos* or atherioscler* or athero-sclero* or atheroma* or arterioscler* or arterio-scler* or atherogene* or athero-gene* or atheros* or microatheros* or fibroatheroma* or fatty streak* or scleros* or cardioscleros* or bifurcation lesion* or occlus* or obstruct* or stenos* or restenos* or syndrome*)).tw,kf.

32 (Bland adj3 Garland adj3 White adj3 syndrome*).tw,kf.

33 ((allergic angina or Kounis) adj3 syndrome*).tw,kf.

34 no-reflow-phenomenon.tw,kf.

35 (CAD or ASCVD).ti,ab.

36 or/6-35

37 5 and 36

**Cochrane Library**

#1 ((maternal or maternity or perinatal or postnatal or post-natal or puerperal or puerperium or post-partum or postpartum or peri-natal) near/3 (mental or depression or depressed or sadness or anxious or anxiet* or coping or panic* or stress* or distress* or psychos* or psych* or PTSD)):ti,ab

#2 ((heart or cardiac or atrial-sept* or atrium-sept* or interatrial-sept* or inter-atrial-sept* or ventricle-sept* or ventricular-sept* or interventricular-sept*) near/3 (anomal* or defect* or malform* or ASD or perforat*)):ti,ab OR ((ductus or truncus) near/3 (arteriosus or arteriosis or botalli) near/3 (patenc* or persisten* or patent* or obliterat* or closure* or ligat*)):ti,ab or ((persist* or patent*) near/3 ostium near/3 secundum):ti,ab OR ((atrial or interventricular or ventricul*) near/3 stunt*):ti,ab or (cleft-heart-atrium* or Lutembacher* or membranous incomplete septum* or arrhythmi* or arrhytmi* or arrythmi* or disarrhythmi* or disarrhytmi* or disarrythmi* or dysarrhythmi* or dysarrhytmi* or dysarrythmi* or "Ehlers Danlos"):ti,ab or (aort* near/3 (coarctatio* or dissect* or ruptur* or dilat*)):ti,ab or (fallot near/3 (tetralogy or triology)):ti,ab or ((Mitral or bicuspid or "left atrioventricular" or aort*) near/3 (disease* or stenosis)):ti,ab or (ectopic near/3 (rhythm* or beat*)):ti,ab or ((heart or cardiac) near/3 (rhythm or beat) near/3 (problem* or disease* or disorder*)):ti,ab or (Heart near/3 (aberrant or ventricle) near/3 (conduct* or contract*)):ti,ab or ((connective tissue* or collagen*) near/3 (defect* or disease* or disorder* or dysplasi*)):ti,ab or ((mesenchymal or sharp* or Weissenbach or Sjoegren or Sjogren or Sicca or "mikulicz radecki" or sacks) near/3 (disorder* or disease* or syndrome*)):ti,ab or (CTD or MCTD):ti,ab or (Marfan* near/3 (abiotroph* or syndrome* or disease*)):ti,ab or ((pulmonar* or lung or portopulmonar* or porto-pulmonar*) near/3 (hypertens* or hemangiomatos* or haemangiomatos* or venoocclusive)):ti,ab or ("cor pulmonale" or corpulmonale or eisenmenger* or aortopath*):ti,ab or (right-heart near/3 failure*):ti,ab or ((pulmonar* or lung) near/3 vascular near/3 (disease* or disorder* or failure*)):ti,ab or (cardiomyopath* or heart myopath* or cardiac myopath* or myocardiopath* or primary myocardial disease*):ti,ab or ((apical or familial or genetic or idiopathic or primary or non-obstructive or nonobstructive or obstructive) near/3 (HCM or HCMP)):ti,ab or ((Beckwith or Weidemann or Costello or Danon or Fabry or Friedreich or LEOPARD or MELAS or MERRF or Noonan) near/3 (syndrome* or disease*)):ti,ab or ((heart or cardiac or myocardial or cardiovascular or cardiogenic or cardiocirculatory or cardio-circulatory or cardiopulmonary or cardiorespiratory) near/3 (failure* or decompensat* or incompeten* or insufficien* or stand-still or standstill or shock or edema* or oedema*)):ti,ab or ((diastolic or systolic or heart or ventricular or ventricle) near/3 (strain or overload or dysfunct*)):ti,ab or ((LV or LVOT or RV or RVOT) near/3 obstruct*):ti,ab or (coronary near/3 (disease* or syndrome* or aneurysm* or anomal* or malformat* or ectasia* or fistula* or calcificat* or calcified or constrict* or vasoconstrict* or spasm* or dissect* or perforat* or thrombos* or atherioscler* or athero-sclero* or atheroma* or arterioscler* or arterio-scler* or atherogene* or athero-gene* or atheros* or microatheros* or fibroatheroma* or fatty streak* or scleros* or cardioscleros* or bifurcation lesion* or occlus* or obstruct* or stenos* or restenos* or syndrome*)):ti,ab

#3 #1 and #2

The Cochrane Library database includes:

1. Cochrane Database of Systematic Reviews
2. Cochrane Central Register of Controlled Trials
3. Cochrane Clinical Answers

**Google Scholar (Via Harzig’s Publish or Perlish)**

postpartum depression cardiovascular disease

**Scopus**

( TITLE-ABS-KEY ( ( maternal OR maternity OR perinatal OR postnatal OR post-natal OR puerperal OR puerperium OR post-partum OR postpartum OR peri-natal ) W/3 ( mental OR depression OR depressed OR sadness OR anxious OR anxiet* OR coping OR panic* OR stress* OR distress* OR psych* OR PTSD ) ) ) AND TITLE-ABS-KEY ( ( heart OR cardiac OR atrial-sept* OR atrium-sept* OR interatrial-sept* OR inter-atrial-sept* OR ventricle-sept* OR ventricular-sept* OR interventricular-sept* ) W/3 ( anomal* OR defect* OR malform* OR asd OR perforat* ) ) OR TITLE-ABS-KEY ( ( ductus OR truncus ) W/3 ( arteriosus OR arteriosis OR botalli ) W/3 ( patenc* OR persisten* OR patent* OR obliterat* OR closure* OR ligat* ) ) OR TITLE-ABS-KEY ( ( persist* OR patent* ) W/3 ostium W/3 secundum ) OR TITLE-ABS-KEY ( ( atrial OR interventricular OR ventricul* ) W/3 stunt* ) OR TITLE-ABS-KEY ( cleft-heart-atrium* OR lutembacher* OR "membranous incomplete septum*" OR arrhythmi* OR arrhytmi* OR arrythmi* OR disarrhythmi* OR disarrhytmi* OR disarrythmi* OR dysarrhythmi* OR dysarrhytmi* OR dysarrythmi* OR "Ehlers Danlos" ) OR TITLE-ABS-KEY ( aort* W/3 ( coarctatio* OR dissect* OR ruptur* OR dilat* ) ) OR TITLE-ABS-KEY ( fallot W/3 ( tetralogy OR triology ) ) OR TITLE-ABS-KEY ( ( mitral OR bicuspid OR "left atrioventricular" OR aort* ) W/3 ( disease* OR stenosis ) ) OR TITLE-ABS-KEY ( ectopic W/3 ( rhythm* OR beat* ) ) OR TITLE-ABS-KEY ( ( heart OR cardiac ) W/3 ( rhythm OR beat ) W/3 ( problem* OR disease* OR disorder* ) ) OR TITLE-ABS-KEY ( heart W/3 ( aberrant OR ventricle ) W/3 ( conduct* OR contract* ) ) OR TITLE-ABS-KEY ( ( "connective tissue*" OR collagen* ) W/3 ( defect* OR disease* OR disorder* OR dysplasi* ) ) OR TITLE-ABS-KEY ( ( mesenchymal OR sharp* OR weissenbach OR sjoegren OR sjogren OR sicca OR "mikulicz radecki" OR sacks ) W/3 ( disorder* OR disease* OR syndrome* ) ) OR TITLE-ABS-KEY ( ctd OR mctd ) OR TITLE-ABS-KEY ( marfan* W/3 ( abiotroph* OR syndrome* OR disease* ) ) OR TITLE-ABS-KEY ( ( pulmonar* OR lung OR portopulmonar* OR porto-pulmonar* ) W/3 ( hypertens* OR hemangiomatos* OR haemangiomatos* OR venoocclusive ) ) OR TITLE-ABS-KEY ( "cor pulmonale" OR corpulmonale OR eisenmenger* OR aortopath* ) OR TITLE-ABS-KEY ( right-heart W/3 failure* ) OR TITLE-ABS-KEY ( ( pulmonar* OR lung ) W/3 vascular W/3 ( disease* OR disorder* OR failure* ) ) OR TITLE-ABS-KEY ( cardiomyopath* OR "heart myopath*" OR "cardiac myopath*" OR myocardiopath* OR "primary myocardial disease*" ) OR TITLE-ABS-KEY ( ( apical OR familial OR genetic OR idiopathic OR primary OR non-obstructive OR nonobstructive OR obstructive ) W/3 ( hcm OR hcmp ) ) OR TITLE-ABS-KEY ( ( beckwith OR weidemann OR costello OR danon OR fabry OR friedreich OR leopard OR melas OR merrf OR noonan ) W/3 ( syndrome* OR disease* ) ) OR TITLE-ABS-KEY ( ( heart OR cardiac OR myocardial OR cardiovascular OR cardiogenic OR cardiocirculatory OR cardio-circulatory OR cardiopulmonary OR cardiorespiratory ) W/3 ( failure* OR decompensat* OR incompeten* OR insufficien* OR stand-still OR standstill OR shock OR edema* OR oedema* ) ) OR TITLE-ABS-KEY ( ( diastolic OR systolic OR heart OR ventricular OR ventricle ) W/3 ( strain OR overload OR dysfunct* ) ) OR TITLE-ABS-KEY ( ( lv OR lvot OR rv OR rvot ) W/3 obstruct* ) OR TITLE-ABS-KEY ( coronary W/3 ( disease* OR syndrome* OR aneurysm* OR anomal* OR malformat* OR ectasia* OR fistula* OR calcificat* OR calcified OR constrict* OR vasoconstrict* OR spasm* OR dissect* OR perforat* OR thrombos* OR atherioscler* OR athero-sclero* OR atheroma* OR arterioscler* OR arterio-scler* OR atherogene* OR athero-gene* OR atheros* OR microatheros* OR fibroatheroma* OR "fatty streak*" OR scleros* OR cardioscleros* OR "bifurcation lesion*" OR occlus* OR obstruct* OR stenos* OR restenos* OR syndrome* ) )

**Web of Science Core Collection**

#1 TS=((maternal or maternity or perinatal or postnatal or post-natal or puerperal or puerperium or post-partum or postpartum or peri-natal) near/3 (mental or depression or depressed or sadness or anxious or anxiet* or coping or panic* or stress* or distress*))

#2 TS=((heart or cardiac or atrial-sept* or atrium-sept* or interatrial-sept* or inter-atrial-sept* or ventricle-sept* or ventricular-sept* or interventricular-sept*) near/3 (anomal* or defect* or malform* or ASD or perforat*)) OR TS=((ductus or truncus) near/3 (arteriosus or arteriosis or botalli) near/3 (patenc* or persisten* or patent* or obliterat* or closure* or ligat*)) or TS=((persist* or patent*) near/3 ostium near/3 secundum) OR TS=((atrial or interventricular or ventricul*) near/3 stunt*) or TS=(cleft-heart-atrium* or Lutembacher* or "membranous incomplete septum*" or arrhythmi* or arrhytmi* or arrythmi* or disarrhythmi* or disarrhytmi* or disarrythmi* or dysarrhythmi* or dysarrhytmi* or dysarrythmi* or "Ehlers Danlos") or TS=(aort* near/3 (coarctatio* or dissect* or ruptur* or dilat*)) or TS=(fallot near/3 (tetralogy or triology)) or TS=((Mitral or bicuspid or "left atrioventricular" or aort*) near/3 (disease* or stenosis)) or TS=(ectopic near/3 (rhythm* or beat*)) or TS=((heart or cardiac) near/3 (rhythm or beat) near/3 (problem* or disease* or disorder*)) or TS=(Heart near/3 (aberrant or ventricle) near/3 (conduct* or contract*)) or TS=(("connective tissue*" or collagen*) near/3 (defect* or disease* or disorder* or dysplasi*)) or TS=((mesenchymal or sharp* or Weissenbach or Sjoegren or Sjogren or Sicca or "mikulicz radecki" or sacks) near/3 (disorder* or disease* or syndrome*)) or TS=(CTD or MCTD) or TS=(Marfan* near/3 (abiotroph* or syndrome* or disease*)) or TS=((pulmonar* or lung or portopulmonar* or porto-pulmonar*) near/3 (hypertens* or hemangiomatos* or haemangiomatos* or venoocclusive)) or TS=("cor pulmonale" or corpulmonale or eisenmenger* or aortopath*) or TS=(right-heart near/3 failure*) or TS=((pulmonar* or lung) near/3 vascular near/3 (disease* or disorder* or failure*)) or TS=(cardiomyopath* or "heart myopath*" or "cardiac myopath*" or myocardiopath* or "primary myocardial disease*") or TS=((apical or familial or genetic or idiopathic or primary or non-obstructive or nonobstructive or obstructive) near/3 (HCM or HCMP)) or TS=((Beckwith or Weidemann or Costello or Danon or Fabry or Friedreich or LEOPARD or MELAS or MERRF or Noonan) near/3 (syndrome* or disease*)) or TS=((heart or cardiac or myocardial or cardiovascular or cardiogenic or cardiocirculatory or cardio-circulatory or cardiopulmonary or cardiorespiratory) near/3 (failure* or decompensat* or incompeten* or insufficien* or stand-still or standstill or shock or edema* or oedema*)) or TS=((diastolic or systolic or heart or ventricular or ventricle) near/3 (strain or overload or dysfunct*)) or TS=((LV or LVOT or RV or RVOT) near/3 obstruct*) or TS=(coronary near/3 (disease* or syndrome* or aneurysm* or anomal* or malformat* or ectasia* or fistula* or calcificat* or calcified or constrict* or vasoconstrict* or spasm* or dissect* or perforat* or thrombos* or atherioscler* or athero-sclero* or atheroma* or arterioscler* or arterio-scler* or atherogene* or athero-gene* or atheros* or microatheros* or fibroatheroma* or fatty streak* or scleros* or cardioscleros* or bifurcation lesion* or occlus* or obstruct* or stenos* or restenos* or syndrome*))

The Core Collection included in this review is:

1. Science Citation Index Expanded (1900 - Data Searched)
2. Social Sciences Citation Index (1900 - Date Searched)
3. Art & Humanities - (1975 - Date Searched)
4. Conference Proceedings Citation Index - Science (1991 - Date Searched)
5. Conference Proceedings Citation Index - Social Sciences and Humanities (1991 - Date Searched)
6. Book Citation Index - Science (2005 - Date Searched)
7. Book Citation Index - Social Sciences and Humanities ( 2005 - Date Searched)
8. Emerging Source Citation Index - (2018 - Date Searched)
9. Current Chemical Reactions (1985 - Date Searched)
10. Index Chemicus (1993 - Date Searched)

**Supplementary Table 3: Excluded Studies Table**

| **Studies Excluded from the Search** | | | | |
| --- | --- | --- | --- | --- |
| **First Authors Last Name** | **Year** | **Title** | **Journal** | **Reason for Exclusion** |
| Aker | 2022 | Perinatal Complications as a Mediator of the Association Between Chronic Disease and Postpartum Mental Illness | Journal of Women’s Health | No pre-existing cardiac conditions |
| Donnenwirth | 2020 | Post-traumatic stress, depression, and quality of life in women with peripartum cardiomyopathy | MCN: The American Journal of Maternal/Child Nursing | No pre-existing cardiac conditions |
| Hutchens | 2022 | Cardiac disease in pregnancy and the first year postpartum: a story of mental health, identity and connection | BMC Pregnancy Childbirth | No effect size/prevalence of post-pregnancy depression, anxiety, or PTSD |
| Liu | 2024 | Postpartum quality of life and mental health in women with heart disease: Integrated clinical communication and treatment | World Journal of Psychiatry | No pre-existing cardiac conditions |
| Pearce | 2023 | Childbearing with Hypermobile Ehlers–Danlos Syndrome and Hypermobility Spectrum Disorders: A Large International Survey of Outcomes and Complications | International Journal of Environmental Research and Public Health | No pre-existing cardiac conditions |
| Pfeffer | 2020 | Assessment of major mental disorders in a German peripartum cardiomyopathy cohort | ESC Health Failure | No pre-existing cardiac conditions |
| Rosman | 2017 | Development of the peripartum cardiomyopathy quality of life registry.DP - 2017 | Dissertation Abstracts International: Section B: The Sciences and Engineering | No pre-existing cardiac conditions |
| Rosman | 2019 | Psychosocial adjustment and quality of life in patients with peripartum cardiomyopathy | Journal of Cardiovascular Nursing | No effect size/prevalence of post-pregnancy depression, anxiety, or PTSD |
| **Studies Excluded from the Search** | | | | |
| **First Authors Last Name** | **Year** | **Title** | **Journal** | **Reason for Exclusion** |
| Brown | 2019 | Chronic physical conditions and risk for perinatal mental illness: A population-based retrospective cohort study | PLoS Medicine | Wrong study design |

| **Table 1. Characteristics of included studies** | | | | | | | | | | |
| --- | --- | --- | --- | --- | --- | --- | --- | --- | --- | --- |
| **First author (year) country** | **Aim** | **Study design** | **CVD classification** | **Sample** | **Age, years** | **Mental health outcomes** | **Time of mental health assessment** | **Instruments** | **Mental health outcome prevalence** | **Association(s) and additional data** |
| Katon (2014) United States | “To examine sociodemographic factors, pregnancy-associated psychosocial stress and depression, health risk behaviors, pre-pregnancy medical and psychiatric illness, pregnancy-related illnesses, and birth outcomes as risk factors for PPD.” | Prospective cohort study | “Pre-pregnancy heart conditions”  May include congenital or acquired conditions as article does not specify | Total study population  n=1,423  Pre-pregnancy heart conditions  n=67 (4.7%) | Mean ± SD = 31.5 ± 5.9 in total study population | Positive PPD screen | 6-week postpartum follow-up | PHQ-9 ≥ 10 | 10.4% (n=7) of pre-pregnancy heart conditions sample screened positive for PPD^1^ | Pre-pregnancy heart conditions not associated with positive PPD screen in multivariate model (OR 1.92, 95% CI 0.98-3.79, p=0.06) |
| Andrade (2020) United States | “To describe the rate of, and risk factors for PPD in women with heart disease.” | Multicenter retrospective cohort study | “Congenital heart disease, acquired valve disease or connective  tissue disease” | CVD only study population  n=129 (100%) | Not reported | PPD | Not specified by authors | Not specified by authors | 19% (n=25) of sample screened positive for PPD (95% CI: 0.13-0.2) | No association between WHO risk score and PPD in multivariate model (aOR 0.7, 95% CI 0.4-1.3)  Prior diagnosis of depression associated with PPD in multivariate model (aOR 8.8, 95% CI 3.3-25.2) |
| Hutchens (2022) Australia | “To explore the general and health-related QOL and mental health outcomes for women who have experienced cardiac disease in pregnancy and the first 12 months postpartum” | Exploratory descriptive study | “A cardiac disease during any pregnancy or in the first 12 months postpartum”  Acquired, congenital and genetic conditions^2^ | CVD only study population n=43 (100%) | Mean age at the time of first cardiac disease in pregnancy or postpartum = 31.39  Range 19– 39 | Positive depression screen  Positive anxiety screen  Cardiac-related anxiety symptoms | At time of assessment  Mean time since first cardiac disease in pregnancy or postpartum was 4.9 years | DASS-21 (no cut-off specified by authors)  CAQ (has no validated clinical cut-off score) | Of those with CHD (n=18):  14% screened positive for depression  22% screened positive for anxiety | Of those with pre-existing CVD^3^ (n=14):  CAQ lowest sub-scale scores were for fear and avoidance; highest sub-scale score was for heart-focused attention |
| **First author (year) country** | **Aim** | **Study design** | **CVD classification** | **Sample** | **Age, years** | **Mental health outcomes** | **Time of mental health assessment** | **Instruments** | **Mental health outcome prevalence** | **Association(s) and additional data** |
| Freiberger (2022) Germany | “The aim of this study was to depict the mental status of postpartum women with CHD in terms of emotional distress and QOL, to explore the concept of illness identity, and to investigate possible medical predictors associated with postpartum mental outcomes.” | Retrospective cross sectional study | “Confirmed diagnosis of congenital heart disease” | CVD only study population  n=121  (100%) | Mean ± SD = 42.7 ± 9.2  Range 27-81 | Positive PPD screen  Positive PTSD screen  Positive anxiety screen | At time of assessment and retrospectively to 6 months postpartum an average of 10.7 years since most recent delivery (SD 10.5, range 0-51) | Mean ± SD scores  EPDS: 7.65±5.9  IES-R: 17.28± 16.60  HADS: 8.93±6.44  Screening tool cut-offs not specified by authors | 6 months postpartum, assessed retrospectively:  32.2% (n=39) of sample screened positive for PPD per EPDS  26.4% (n=32) of sample screened positive for mild PTSD per IES-R  18.2% (n=22) of sample screened positive for mild PTSD per IES-R | No correlation between WHO risk score and positive PPD screen (rs=0.148)  Significant correlation between WHO risk score and positive PTSD screen (rs=0.209, p=0.031)  Significant differences in IES-R score between WHO risk score I and IV (p=0.012), linear regression found higher WHO risk score associated with higher IES-R score (p=0.015) |
|  |  |  |  |  |  |  |  |  | At time of assessment: 14.9% (n=18) of sample screened positive for depression per HADS  24.8% (n=30) of sample screened positive for anxiety per HADS | No correlation between WHO risk score and positive depression screen (rs=0.005) or positive anxiety screen (rs=0.022) |
| **First author (year) country** | **Aim** | **Study design** | **CVD classification** | **Sample** | **Age, years** | **Mental health outcomes** | **Time of mental health assessment** | **Instruments** | **Mental health outcome prevalence** | **Association(s) and additional data** |
| Panelli (2022) United States | “To evaluate clinical factors associated with a positive PPD screen in pregnant people with cardiac disease” | Retrospective cohort study | “Congenital and/or acquired cardiac disease during pregnancy” | CVD on study population n=126 (100%) | Median age at delivery (Q1, Q3) = 33.1 (29.4, 36.6) | Positive PPD screen | Median weeks postpartum (Q1, Q3) = 6 (6, 7) | EPDS≥ 10 | 18.3% (n=23) of sample screened positive for PPD  15% (n=10) of those with acquired disease vs. 20% (n=12) of those with CHD screened positive for PPD^1^  20.6% (n=26) of sample with pre-pregnancy depression or anxiety; neither significantly associated with positive PPD screen (p=0.78) | Trend towards higher WHO score amongst those who screened positive for PPD, though not significant (p=0.17)^4^  Participants with positive PPD screen more likely to have had antepartum anticoagulation (p=0.007), blood transfusion (p=0.032) and maternal-infant postpartum separation (p=0.047). |
| Zipursky (2022) Canada | “To examine the association between ECG testing in pregnancy and PPD” | Cohort study | “Heart disease” (ICD-9 Codes  390-429) | Total sample  n=3,238,218  Total ECG cohort  n=157,352  Total with heart disease n=86,587 | Median age of total study population = 30 | Physician diagnosis of PPD | Within 1 year postpartum | ICD-9 Code 311^5^, required physician contact for depression diagnosis on two separate occasions in the first year postpartum^6^ | 1.48% (n=48,047) with PPD diagnosis in total sample  No prevalence data for PPD diagnosis in heart disease sample | History of heart disease not associated with increased odds of PPD diagnosis in multivariate model (aOR 1.05, 95% CI 1.00-1.11) |

| **First author (year) country** | **Aim** | **Study design** | **CVD classification** | **Sample** | **Age, years** | **Mental health outcomes** | **Time of mental health assessment** | **Instruments** | **Mental health outcome prevalence** | **Association(s) and additional data** |
| --- | --- | --- | --- | --- | --- | --- | --- | --- | --- | --- |
| Abu-Zaid (2023) United States | “To investigate the association between different chronic medical conditions and mental illnesses during pregnancy and the peripartum periods using the NIS” | Retrospective cohort study | Heart disease (ICD-10 Code not reported)  Previous heart failure^7^  Atrial fibrillation (ICD-10 Code I48*) | Total sample  n=2,854,149  Heart disease n=1,810  Heart failure n=374  Atrial fibrillation n=959 | Mean age of total sample ± SD = 29 ± 6 | Hospital admissions for PPD | Within 1 year postpartum | PPD (ICD-10 Code F530) | 0.042% (n=1,105) of total sample admitted for PPD^1^  0.17% (n=3) of those with heart disease admitted for PPD^1^  0.21% (n=3) of those with atrial fibrillation admitted for PPD^1^ | No significant associations between CVD variables and admission for PPD in multivariate models  Heart disease: (OR 0.8, 95% CI 0.0-17.0, p=0.865)  Previous heart failure:  (OR 6.9 95% CI 0.3-158.0 p=0.225)  Atrial fibrillation: (OR 4.6, 95% CI 0.2-103.1, p=0.339) |

Abbreviations: Postpartum depression (PPD), standard deviation (SD), Patient Health Questionnaire (PHQ-9), odds ratio (OR), cardiovascular disease (CVD), World Health Organization (WHO), congenital heart disease (CHD), quality of life (QOL), post-traumatic stress disorder (PTSD), Edinburgh Postnatal Depression Scale (EPDS), Impact of Events Scale Revised (IES-R), Hospital Anxiety and Depression Scale (HADS), Depression Anxiety and Stress Scale (DASS-21), Cardiac Anxiety Questionnaire (CAQ), International Classification of Diseases (ICD), National Inpatient Sample (NIS)

^1^ Calculated from author reported information

^2^ Authors specify conditions include cardiomyopathies, rhythm disorders, coronary artery dissection and myocardial infarction, structural anomalies and valvular conditions. Primary diagnosis of hypertension or preeclampsia excluded

^3^ Includes congenital heart disease and acquired heart disease.

^4^ Of those with positive PPD screen (n=23), 26% in class I, 35% with class II, 35% with class III, and 4% with class IV; of those without PPD (n=103) 38% with class I, 37% with class II, 12% with class III, 8% with class IV

^5^ Excluded psychotic disorders, substance abuse, other psychiatric diagnoses

^6^ Specificity 94%, sensitivity 61%, positive predictive value 70%, negative predictive value 92%)

^7^ ICD-10 Codes: I50.22, I50.23, I50.32, I50.33, I50.42, I50.43, I50.812, I50.813
